# Hereditary Prostate Cancer: Genes Related, Target Therapy and Prevention

**DOI:** 10.3390/ijms22073753

**Published:** 2021-04-04

**Authors:** Maria Teresa Vietri, Giovanna D’Elia, Gemma Caliendo, Marianna Resse, Amelia Casamassimi, Luana Passariello, Luisa Albanese, Michele Cioffi, Anna Maria Molinari

**Affiliations:** 1 Department of Precision Medicine, University of Campania “Luigi Vanvitelli”, Via L. De Crecchio, 80138 Naples, Italy; amelia.casamassimi@unicampania.it (A.C.); annamaria.molinari@unicampania.it (A.M.M.); 2 U.O.C. Clinical and Molecular Pathology, A.O.U. University of Campania “Luigi Vanvitelli”, 80138 Naple, Italy; giovanna.delia@policliniconapoli.it (G.D.); gemma.caliendo@unicampania.it (G.C.); marianna.resse@unicampania.it (M.R.); luana.passariello@studenti.unicampania.it (L.P.); luisa.albanese@studenti.unicampania.it (L.A.); michele.cioffi@unicampania.it (M.C.)

**Keywords:** hereditary prostate cancer, genetic testing, genotype–phenotype correlation, surveillance

## Abstract

Prostate cancer (PCa) is globally the second most diagnosed cancer type and the most common cause of cancer-related deaths in men. Family history of PCa, hereditary breast and ovarian cancer (HBOC) and Lynch syndromes (LS), are among the most important risk factors compared to age, race, ethnicity and environmental factors for PCa development. Hereditary prostate cancer (HPCa) has the highest heritability of any major cancer in men. The proportion of PCa attributable to hereditary factors has been estimated in the range of 5–15%. To date, the genes more consistently associated to HPCa susceptibility include mismatch repair (MMR) genes (*MLH1*, *MSH2*, *MSH6*, and *PMS2*) and homologous recombination genes (*BRCA1/2*, *ATM*, *PALB2*, *CHEK2*). Additional genes are also recommended to be integrated into specific research, including *HOXB13*, *BRP1* and *NSB1*. Importantly, *BRCA1/BRCA2* and *ATM* mutated patients potentially benefit from Poly (ADP-ribose) polymerase PARP inhibitors, through a mechanism of synthetic lethality, causing selective tumor cell cytotoxicity in cell lines. Moreover, the detection of germline alterations in MMR genes has therapeutic implications, as it may help to predict immunotherapy benefits. Here, we discuss the current knowledge of the genetic basis for inherited predisposition to PCa, the potential target therapy, and the role of active surveillance as a management strategy for patients with low-risk PCa. Finally, the current PCa guideline recommendations are reviewed.

## 1. Introduction

Prostate cancer (PCa) is globally the second most diagnosed cancer type in men [1] and the most common cause of cancer-related deaths, with an estimated 1,600,000 cases and 366,000 deaths annually [2]. Established risk factors include older age, African American race, and a positive family history of PCa [3].

PCa is clinically a very heterogeneous disease; indeed, many patients show an aggressive disease with progression and metastasis while other patients show a slow disease with low propensity to progression [2]. Histologically, these tumors are measured in terms of the Gleason score that evaluates how much the biotic prostatic specimen is similar to the normal prostate gland [2].

Compared to sporadic cases, HPCa is characterized by an early age onset, an aggressive disease progress and locally advanced stage. Furthermore, men with HPCa have a higher risk of recurrence after surgery, while there is not much difference between HPCa and sporadic PCa regarding overall survival [4].

Family history of PCa, hereditary breast and ovarian cancer (HBOC) syndrome and Lynch syndrome (LS) are among the most important risk factors compared to age, race, ethnicity and environmental factors for the development of PCa [5,6,7,8,9] and this risk is estimated at 40%−50 % [3]. Men with a brother or father diagnosed with prostate cancer have a two- to four-fold greater risk of developing PCa [10].

Hereditary prostate cancer (HPCa) has the highest heritability of any major cancer in men [11]. Since PCa is asymptomatic in the early stage of the disease, it is critical to develop an individualized approach for early detection [12]. Patients with early onset of PCa associated to family members affected with PCa or other heritable cancers are suitable candidates to undergo a genetic testing [6]. Over time, about 170 susceptibility loci for HPCa, accounting for ~33% of familial prostate cancer risks, have been identified with genome wide association studies (GWAS) [13,14]. Further insights have suggested that mutations in the different DNA damage repair (DDR) genes (*BRCA1*, *BRCA2*, *CHEK2*, *ATM* and *PALB2*) and in the DNA mismatch repair genes (MMR) (*MLH1*, *MSH2*, *MSH6* and *PMS2*), are biomarkers of HPCa [6,15,16]. Importantly, while many genes have a clear association with HPCa risk, others carry a still unknown clinical significance with a poorly defined cancer risk [16]. Besides, there is strong emerging evidence that mutation in some genes may predict the response to poly-ADO ribose polymerase (PARP) inhibitors and platinum-based chemotherapy in prostate cancer [17].

Here, we report on the current knowledge of the genetic basis for inherited predisposition to PCa, the potential target therapy, and the role of active surveillance as a management strategy for patients with low-risk PCa. Finally, the current PCa guideline recommendations are reported.

## 2. Epidemiology of Hereditary Prostate Cancer

The proportion of PCa attributable to hereditary factors has been estimated to be 5–15% [6]. HPCa is due to gene mutations with autosomal dominant inheritance and characterized by early onset [18]. Heidegger et al. reported that the cumulative proportion of PCa cases attributable to high-risk susceptibility alleles is 43% for men diagnosed <55 years, but only 9% for men >85 years [6].

HPCa incidence is two and three times higher in African Americans men than European and Asian men, respectively [12,15]. This outcome may be influenced by lifestyles such as diet and obesity, as well as screening patterns within an ethnic/racial community [15]. Likewise, genetic etiology differs among population diversity [6]. For example, single-nucleotide polymorphisms (SNPs) that had been previously reported in white or Asian populations were not found in Afro-American men. In line with these studies, other genome-wide association studies (GWAS) did not replicate most of the previously reported loci identified in European or Asian descent populations [6]. Accordingly, a novel locus on chromosome 10p14 (SNP, rs7918885), exclusively detected in African men, has been described [6]. This SNP, rs7918885, is localized at 10p14 within an intron of a long non-coding RNA (lncRNA RP11-543F8.2) 360 kb centromeric of GATA3 [19]. Additionally, several *BRCA1/2* variants of unknown significance (VUS) were observed in African American patients more frequently than in Caucasian Americans (4.6 % vs. 1.6 %, respectively) [1].

On the other hand, there is one specific chromosomal locus at 8q24 (near the MYC proto-oncogene) where several SNPs have been associated with PCa susceptibility among various ethnicities [15].

## 3. Genes Involved in the Predisposition to Hereditary Prostate Cancer

HPCa susceptibility loci were found on all chromosomes except 15, 16, 21, and 23 [11].

**Figure 1:**
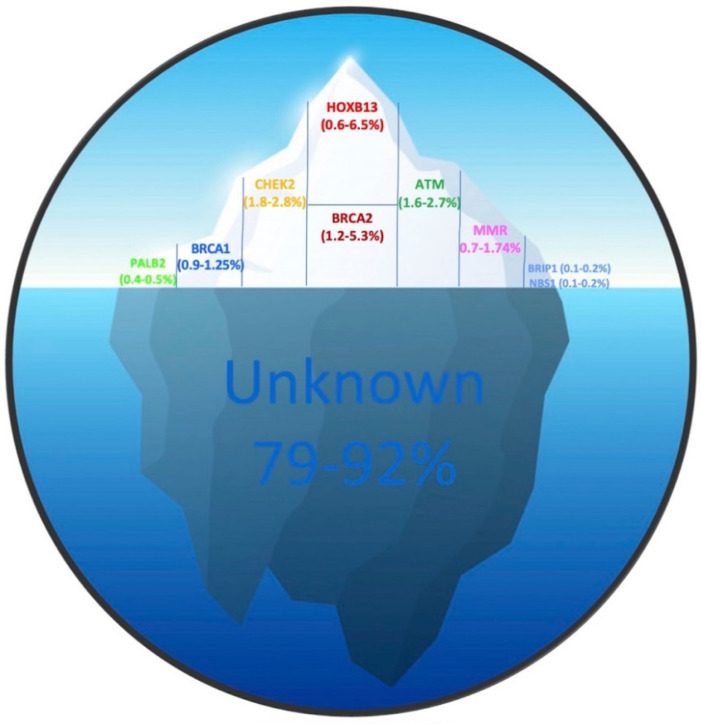
Frequency of mutations found in HPCa related genes. The proportion of PCa attributable to hereditary factors has been estimated to be 5–15%. The upper part of the iceberg constitutes 7.4–21% and indicates the gene mutation frequencies known so far, while the lower part, constituting 79–92%, represents the portion of genes not yet identified. Partner and localizer of BRCA2 (*PALB2*); Breast cancer type1 (*BRCA1*); Checkpoint kinase 2 (*CHEK2*); Breast cancer type2 (*BRCA2*); Homeobox B13 (*HOXB13*); ATM serine/threonine kinase (*ATM*); Mismatch Repair (MMR); BRCA1 interacting protein C-terminal helicase 1 (*BRIP1*); Nijmegen Breakage Syndrome 1 (*NBS1*).

In 0.6–6.25% of cases HPCa is due to *HOXB13* mutations, in 1.2–5.3% to *BRCA2*, in 1.8–2.8% to *CHECK2*, in 1.6–2.7% to *ATM*, in 0.7–1.74% to *MMR*, in 0.9–1.25% to *BRCA1*, in 0.4–0.5% to *PALB2*, in 0.1–0.2% to *BRP1* and *NBS1* and in the remaining percentage to other currently unknown genes (Figure 1) [13,15,18,20,21].

To date, the genes more consistently associated to HPCa susceptibility have been reported by the National Comprehensive Cancer Network (NCCN) guidelines and include LS associated genes (*MLH1*, *MSH2*, *MSH6*, and *PMS2*) and homologous recombination genes (*BRCA1/2*, *ATM*, *PALB2*, *CHEK2*) [22]. Additional genes, including the above mentioned *HOXB13, BRP1* and *NSB1*, are also recommended in specific research or clinical contexts.

*HOXB13* is a homeobox transcription factor, localized on chromosome 17. Mutations have been observed in 0.7% to 1.4% cases of prostate cancer and in 6% of PCa with early onset [23]. In 2012, a study demonstrated for the first time that patients with the recurring germline mutation G84E in *HOXB13* had significantly higher odds for developing PCa than men without the mutation [24]. Subsequent studies have confirmed a more modest association with an increased risk of PCa, particularly in the hereditary setting [25]. In a more recent study, *HOXB13* mutation has been associated with an increased HPCa risk (OR = 6.6) [6]. To date, *HOXB13* remains the most widely replicated and specific PCa susceptibility gene [26].

It is well known that *BRCA* genes are considered as the principal biomarkers for the genetic study of hereditary breast and ovarian cancers [27]; however, several authors have also reported an association in patients affected with HPCa, which is part of HBOC syndrome [8,15,28]. *BRCA1* and *BRCA2* are tumor suppressor genes, localized on chromosomes 17 and 13, respectively, involved in repairing DNA double strand breaks by the conservative approach of homologous recombination (HR) [29]. The germline *BRCA1* mutations increase the risk of HPCa by 3.8-fold in men aged <65 years, and germline *BRCA2* mutations by 8.6-fold [8]. In a study performed on a cohort of 2019 HPCa patients, 0.9% germline mutations in *BRCA1* and 3% in *BRCA2* were observed [30]. Moreover, Edwards et al. reported a median overall survival (OS) of all *BRCA2* mutation carriers of 4.8 years vs. 8.5 years for non-carriers [28]. Additionally, as expected, in isolated and/or consanguineous populations, heterozygous carriers of one founder *BRCA* mutation exhibited increased susceptibility to HPCa [28]. Otherwise, some authors have reported different data. Indeed, Rantapero et al. described a frequency of zero cases of pathogenic *BRCA2* variants in 122 lethal cases, suggesting that *BRCA2* could not play a major role in aggressive and lethal PCa [14]. Another study has shown that *BRCA2* deletions accounted for a very small number of HPCa (1–2%) even in cases with early onset and proven family history [6].

The *CHEK2* gene, localized on chromosome 22, encodes for a tumor suppressor that participates in the DNA-damage signaling pathway. Alterations in *CHEK2* have been found in HBOC syndrome, with an increased risk of breast, ovarian, colon, thyroid and kidney cancer, and have been linked to Li-Fraumeni syndrome [15]. Germline *CHEK2* mutations have been also associated with increased HPCa risk with an overall risk of 1.9–3.3% [6]. In a study performed on metastatic PCa patients from Sweden *CHEK2* was also the most frequently mutated DNA repair gene (3.8%) highlighting the importance of *CHEK2* mutations for aggressive PCa in the Nordic population [31].

Another gene of interest for HPCa is *ATM*, as recently reviewed in [32]. *ATM* is positioned on chromosome 11 and represents a key player in the DNA damage response pathway. Homozygous loss-of-function mutations in *ATM* cause ataxia telangiectasia syndrome. Of note, it is also one of the known HBOC susceptibility genes; indeed, *ATM* mutation carriers have an increased risk for breast, colorectal, gastric and pancreatic cancers. More recently, the use of sequencing panels containing DNA-repair genes has also allowed the identification of germline ATM alterations in men with PCa. [15]. The relative risk of metastatic prostate cancer in *ATM* carriers was 6.3% [6].

Interestingly, patients with germline mutations in the *MMR* genes, *MLH1*, *MSH2, MSH6* and *PMS2*, located on chromosome 3, 2, 2 and 7, respectively, have a HPCa risk [30,33]. However, the estimated prevalence of *MMR* gene mutations is low when compared to alterations in genes of other DDR pathways [34]. As already known, the *MMR* genes are associated with LS that predisposes to colorectal and endometrial cancer, although several other extracolonic cancers have been reported, including gastric, small bowel, pancreatic, brain, prostate and urothelial [35,36]. The relative risk of HPCa in *MMR* mutation carriers has been estimated in the interval 2.0–3.7% [23], even though a previous study reported a nearly five-fold increased risk of HPCa onset in LS men, but without earlier onset or a more aggressive phenotype [37]. Additionally, studies have also highlighted a considerably higher PCa risk for *MSH2* mutation carriers compared to *MLH1* and *MSH6* [38].

*PALB2* is localized on chromosome 16; originally identified as a *BRCA2*-interacting protein, it is an essential component of the *BRCA* complex formation. Indeed, *PALB2* acts as a bridge between *BRCA1* and *BRCA2* to form a *BRCA* complex that initiates homologous recombination [39]. Various *PALB2* mutations are involved in HBOC predisposition, particularly associated with breast and pancreatic cancers, whereas few studies have reported *PALB2* variants in HPCa patients. In the analysis by Pritchard et al., *PALB2* germline mutations were detected in 0.4% of HPCa cases [18]. Nicolosi reported pathogenic *PALB2* germline variants in 0.5% of unselected PCa patients [18,40]. Apart from the rarity of reported *PALB2* alterations, recent findings have supported an increasing role of *PALB2* in HPCa disease [41].

A few less-studied candidate genes have been proposed to be associated with HPCa predisposition, which are involved in a small percentage of HBOC cases. One of the candidate genes is *BRIP1*. This gene is localized on chromosome 17, encodes a DNA helicase, which binds directly to the BRCT repeats of BRCA1, and is therefore involved in DSB repair [42]. To date, few studies have reported potentially damaging *BRIP1* variants in PCa patients; particularly, Kote-Jarai et al. found a moderate risk of PCa in a set of familial and young-onset PCa patients carrying a recurrent *BRIP1* truncating variant [43].

Another gene of interest is *NBS1*, which is located on chromosome 8 and encodes another protein involved in DSB repair since it is responsible for the localization of the complex and for the interactions with other proteins belonging to repair signaling complexes [44]. A specific founder variant (c.657del5) has been associated with a three-fold increased risk of PCa in patients under 60 years, and a four-fold increased risk for male carriers with a positive family history [45].

Other inherited alterations in genes associated with DNA repair have been identified in HPCa studies, including *RAD51C*, *RAD51D*, and *TP53* genes. Unfortunately, very little data is currently available about the existence of such associations. Thus, further studies with an expanded set of genes are warranted to validate these and other proposed candidates as HPCa susceptibility genes [18,46].

## 4. Pathogenetic Mechanisms of PCa Onset

**Figure 2:**
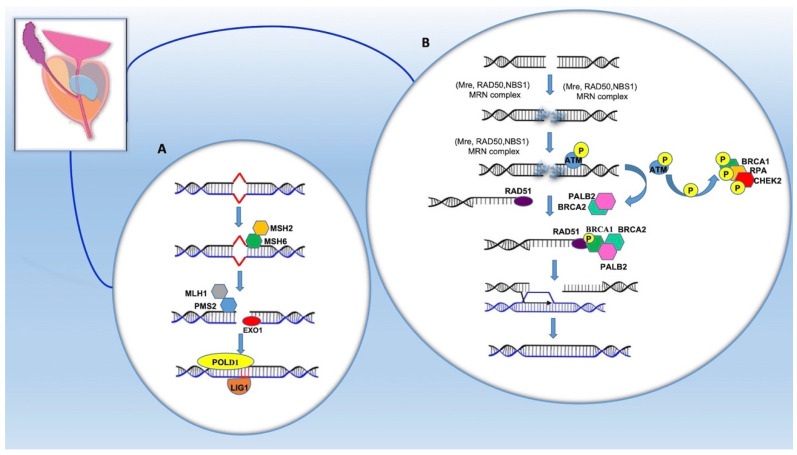
DNA repair pathway in the prostate epithelium involving hereditary prostate cancer (HPCa) related genes: mismatch repair or MMR (**A**); homologous recombination or HR (**B**). See text for detailed description. mutS homolog 2 (MSH2); mutS homolog 6 (MSH6); mutL homolog 1 (MLH1); PMS1 homolog 2 (PMS2); Exonuclease 1 (EXO1); δ polymerase (POLD1); DNA ligase I (LIG1); Meiotic recombination 11 (MRE11); Double strand break repair protein (RAD50); Nijmegen disruption syndrome proteins (NBS1); Replication protein A (RPA); ATM serine/threonine kinase (ATM); Breast cancer type1 (BRCA1); Checkpoint kinase 2 (CHEK2); RAD51 recombinase (RAD51); Breast cancer type2 (BRCA2); Partner and localizer of BRCA2 (PALB2).

Alterations in the DNA damage repair (DDR) pathways have been recently recognized as an important contributor to PCa [17]. The DDR system includes multiple distinct pathways, two of which are: (a) homologous recombination (HR), which relies on BRCA1, BRCA2 and ATM, and (b) mismatch repair (MMR), which involves MLH1, MSH2, MSH6 and PMS2 [47]. Repair of the breaks in the DNA double strand (DSB) requires the intervention of several proteins encoded by genes such as *BRCA1*, *BRCA2*, *ATM*, *RAD51*, *PALB2* and others. These genes are tumor suppressors and some of them cooperate with each other (Figure 2).

HR is activated during the synthesis, or S phase, of the cell cycle, and requires a sister chromatid as a template to repair damaged DNA. If repair does not occur, the damage can lead to deletions, chromosomal aberrations and aneuploidies [48]. 

In the event of damage, the disruption of the DSB is recognized by the poly (ADP-ribose) polymerase 1 (PARP1) complex, a protein that scans the genome and detects DSB lesions [49]. PARP1 marks the site of damage by binding the ribose ADP molecules to chromatin-bound proteins surrounding the break [50]. ADP-ribose units are essential for the recruitment of MRN complex, formed by MRE11 (meiotic recombination 11), RAD50 and NBS1 (Nijmegen disruption syndrome) proteins.

The localization of MRN triggers the signaling of downstream repair factors, mediated by ATM and ATR kinases. ATM is then recruited to the rupture sites by the MRN complex, self-phosphorylates and monomerizes. Once activated, ATM phosphorylates various substrates including BRCA1, the MRN complex and Replication protein A (RPA). Moreover, ATM phosphorylates histone H2AX which induces a signal cascade involving protein mediators such as CHEK2 [34]. The exonuclease activity of MRN complex produces single-stranded DNA (ssDNA) surrounding the break [50,51,52,53]. RPA recognizes ssDNA and recruits ATR, thereby activating an additional cascade that includes ATR substrates such as CHEK1 and other effector proteins. Once activated, ATM phosphorylates various substrates including BRCA1, the MRN complex and RPA. MRN remodels the ends leaving ssDNA to which RAD51 is linked, localized by BRCA2, which assumes a multimeric filamentous structure and mediates the invasion of the helix of the homologous chromosome to look for a homology region with which to repair the damage. PALB2 acts as a bridge between BRCA1 and BRCA2 to form a BRCA complex that then binds RAD51 [54]. Holliday junctions are created, also characteristic of crossing-over; finally, thanks to δ polymerase, the structure is resolved, and the damage is repaired [34].

The MMR pathway recognizes and removes DNA base pair mismatches occurring because of replication errors [55]. Genes encoding for MSH and MLH family proteins are involved in this pathway. These MutSa Complex heterodimers (MSH2/MSH6) and MutLb (MLH1/PMS1) or MutSb (MSH2/MSH3) and MutLa (MLH1/PMS2) bind to the surrounding DNA [56]. Initially, there is the recognition of the mismatch by the heterodimer MSH2/MSH6, followed by the recruitment of MLH1/PMS2. The activity of the PMS2 endonuclease determines synchronous stranded nicks close to the mismatch, where the EXO1 exonuclease interacts with the nuclear antigen of proliferating cells (PCNA), starting the resection of the DNA from 5’ to 3’. Finally, the strand is resynthesized by the δ polymerase (POLD1) and bound by DNA ligase I (LIG1) [56].

Disruption of these DNA repair pathways leads to increased mutagenesis and genomic instability with the consequent accumulation of genomic aberrations [57]. It is established that PCa tissues display recurrent genomic aberrations, including alterations at 8p (containing the prostate-specific tumor suppressor *NKX3–1*), 8q (*c-MYC* amplification), 10q23 (*PTEN* loss), 17q (*TP53* locus) and Xq12 (*AR* gene amplification) as well as *TMPRSS2:ERG* gene fusion or other fusion events involving additional ETS family members [58]. Although a clear relationship between these alterations and germline mutations in HPCa has not yet been demonstrated, they could be, at least in part, due to the disruption of DNA repair pathways. Indeed, PCa tumors with germline *BRCA2* mutations displayed significantly more copy number alterations (CNA) than sporadic tumors. Specifically, the deletion of chromosome 8p was observed as the most recurrent somatic CNA, whereas chromosome 8q alteration, at 8q24.21, was the most frequent gain. Interestingly, this study also revealed that high CNA level was already present in 50% of the morphologically normal prostate tissue from *BRCA2* mutation carriers [59].

Besides, although carried out on metastases from castrate-resistant PCa, a study evidenced a correlation between some alterations of critical tumorigenesis regulators and structural variants. For instance, deletions were significantly higher in tumors with biallelic *BRCA2* pathogenic variants (including germline mutations). Additionally, biallelic *CDK12* inactivation was associated with a significant increase of tandem duplications whereas biallelic *TP53* inactivation was significantly associated with elevated inverted rearrangement frequency. Overall, these data suggest that specific genomic alteration signatures could develop from different germline mutations [60]. 

## 5. Mutations and Genotype–Phenotype Correlation

The G84E mutation of *HOXB13* gene was found to be more prevalent in European families, suggesting a possible founder effect [5,61]. Additional *HOXB13* mutations have also been detected in PCa cases in other racial or ethnic groups, including African (G216C and R229G) and Asian (G135E) populations, but the frequency and impact of these mutations on the PCa risk remains to be confirmed [20]. It has been highlighted that *HOXB13* G84E mutation is associated with early-onset, familial and high-risk of PCa [15,62].

Regarding *BRCA* genes, 64% of the *BRCA2* mutations found in HPCa are frameshift, 31% missense and 5% splice. In *BRCA1* gene, 63% of the mutations found are missense, 31% frameshift and 6% splice [38]. Besides, in a study conducted on PCa patients mutated in *BRCA1/2*, a more aggressive phenotype was observed [30,62].

Remarkably, germline alterations were independent factors of a diagnosis at younger age and of a more aggressive phenotype. Besides, *BRCA2* mutation carriers exhibited a poorer survival rate (61.8%) compared to men without a *BRCA2* mutation (94.3%). *BRCA2* pathogenic variants were also associated with an increased risk of high-grade disease and progression to metastatic castrate resistant prostate cancer (mCRPC). Besides, *BRCA2* germline mutations provided a greater contribution to PCa increased risk compared to *BRCA1* [3,8,11,15,23,28,63].

*BRCA1* mutation carriers younger than 65 years showed a two-fold increase of PCa relative risk, whereas no evidence of an elevated risk in men aged 65 or older was observed. The *BRCA1* Q356R was preferentially transmitted to affected men of white families with early-onset HPCa at an estimated odds ratio of 2.25 [20]. Several studies have evaluated the contribution of *BRCA1* (185delAG and 5382insC) and *BRCA2* (6174delT) founder mutations to PCa risk among Ashkenazi Jewish men. Overall, *BRCA2* mutations conferred at least a three-fold elevated risk of high grade PCa, while *BRCA1* mutations conferred a lower risk [38,64,65].

In the *CHEK2* gene, 44% of the mutations found in HPCa are frameshift, 31% missense and 25% splice. *CHEK2* variants have been associated with PCa predisposition in several studies, highlighting the importance of *CHEK2* mutations for aggressive PCa [14]. Moreover, a significantly higher prevalence of germline alterations in the *CHEK2* gene was observed in the metastatic cases, although no association with age at diagnosis was reported [15]. Of note, the c.1100delC mutation was the most commonly observed *CHEK2* mutation and was found to have a significantly higher frequency in lethal cases compared than in the low-risk PCa patients, supporting the importance of this mutation for lethal PCa [14]. Moreover, the c.1100delC mutation and I157T increased the PCa risk by 3.3% and 1.8%, respectively [15].

As regards the *ATM* gene, 50% of mutations found in HPCa are missense, 37% frameshift and 13% splice. *ATM* variants were associated to aggressive and lethal phenotype of HPCa disease [66]. Recently, *ATM* deficiency has been shown to promote the progression of castration-resistant PCa [14]. Moreover, the *ATM* 3161G (P1054R) variant was significantly associated with an increased risk of developing PCa [20].

Concerning MMR genes, 47% of the mutations found in HPCa are frameshift, 44% missense and 9% splice. In a recent study, the MMR gene mutation carriers with PCa displayed microsatellite instability and loss of MMR gene expression, implicating this pathway in prostate cancer formation. The PCa patients with MMR deficiency showed aggressive clinical and pathological features [67]. Significantly, MMR gene mutation carriers developed PCa in earlier age and at a higher frequency than expected. Moreover, MMR mutation carriers displayed a 3% higher risk of PCa compared to the general population [20].

In *PALB2* gene, 83% of the mutations found in HPCa are frameshift and 17% missense. Despite the rarity of reported PALB2 aberrations, recent findings have supported an increasing role of *PALB2* in PCa, particularly in metastatic cases [68].

Additional genomic studies based on Next-Gen Sequencing (NGS) can allow the identification of other genes potentially involved in HPCa onset. Moreover, future studies on genotype-phenotype correlation would be needed and these studies they should converge in a meta-analysis that could provide a greater understanding of HPCa.

## 6. Therapeutic Target

The therapeutic landscape of PCa is constantly evolving thanks to clinical trial benefits, new therapeutics, use of NGS, advanced functional imaging and the better use of existing therapies in the early-stage disease. PCa initiation and disease progression are driven by AR signaling [69]. It is known that the PCa is unique in its dependence on androgen for growth and progression, and androgen deprivation therapy (ADT) is an effective treatment for patients with advanced disease over 75 years [70]. However, when a castration-resistant state occurs, the patient is more likely to die of PCa than other causes [71]. Alterations in AR signaling in metastatic castration resistant PCa (mCRPC) cause persistent AR activation, which in turn leads to AR amplification, AR splice variants and intra-tumoral androgen biosynthesis [70]. Therapeutic strategies involve the use of enzalutamide, an AR antagonist that blocks AR translocation function, and abiraterone which inhibits androgen biosynthesis [67].

Recently, mCRPC patients with germline defects in DNA damage repair showed a decreased response to AR targeted therapy [6]. In contrast, other authors reported an improved response to second generation ADT with administration of drugs, including abiraterone or enzalutamide, in men with *BRCA* or *ATM* mutations compared to those without deleterious germline mutations [67].

Thus, the mutation status of genes involved in HPCa may have an impact on therapeutic strategies [16,72]. For instance, mutated patients potentially benefit from PARP inhibitors such as Olaparib, rucaparib, niraparib and telazoparib [1,18], through a mechanism of synthetic lethality, causing selective tumor cell cytotoxicity in cell lines. 

The Food and Drug Administration (FDA) approved the drug Olaparib for the treatment of patients with mCRPC, mutated in *BRCA1/BRCA2* and *ATM* genes. In line with previously described results, in patients with germinal *BRCA2* or *ATM* mutations, treatment with the PARP inhibitor Olaparib has a durable antitumor activity [73]. Moreover, based on further studies, PARP inhibitor rucaparib was also added for mCRPC patients with deleterious *BRCA* alterations previously treated with ADT, but not for patients with *ATM* mutations [74]. Besides, other studies have shown that the presence of DDR defects may also be predictive of a higher likelihood of a response to carboplatin-based chemotherapy in patients with mCRPC [75].

The detection of germline alterations in MMR genes also has therapeutic implications, as it may help to predict immunotherapy benefits. Recent studies have suggested that metastatic PCa patients with germline MMR pathogenic variants may have a particular sensitivity to hormonal therapies, as well as a possible response to PD−1 inhibitors [76]. Additional clinical studies reported either a complete or a partial response to PD−1 inhibitors in mCRPC patients [77]. Based on these findings, the FDA has recently approved the PD−1 inhibitor pembrolizumab (KEYTRUDA) for the treatment of patients with microsatellite instability-high (MSI-H)/MMR-deficient [78]. The use of genetically based therapies sustains the importance of applying genetic testing in the clinical management of these PCa patients. 

The PD−1 pathway includes the programmed death protein−1 (PD−1) and its ligands, PD-L1 (B7-H1) and PD-L2 (B7-DC). This pathway has emerged as a mechanism for immune tolerance whereby tumor cells can suppress an antitumor immune response. Taking into account the high aggressivity of PCa, particularly mCRPC, and the success of PD−1/PD-L1 blockade in other cancers, patients with mCRPC could benefit from correct modulation of the immune system and, hence, from the use of checkpoint inhibitors [18]. The activity of the checkpoint inhibitors is limited to a certain percentage of patients, depending on the tumor type. This is especially important in tumors with low objective response rates to immunotherapy, such as PCa. Tumor cell PD-L1 expression is a predictive biomarker for PD−1 inhibitor sensitivity. Examples of subgroups of PCa patients with enriched PD-L1 expression are patients with aggressive tumors and with tumors harboring somatic or germline DDR mutations, including patients with HPCa. The presence of DDR or MMR mutations may favor the activity of checkpoint immunotherapy. Therefore, the use of PD−1/PD-L1 inhibitors for advanced prostate cancer should be encouraged in the setting of clinical trials and to identify the patient subgroups that can benefit from these therapies. Furthermore, it would be interesting and useful to develop a biomarker panel to predict benefit and response of these and other checkpoint inhibitors [78].

Current National Comprehensive Cancer Network (NCCN) guidelines recommend three gene expression-based tests for PCa prognosis in men with low or favorable intermediate risk disease: Decipher, Oncotype DX Prostate, and Prolaris [79]. Particularly, the current recommendations evaluate the role of each of the panels in both prostate biopsy and post-surgery settings. Overall, these tests provide more precise estimates of disease aggressiveness, beyond clinical factors, and they could help to guide on appropriate disease management and therapy. Thus, they could also be used for active surveillance in HPCa. Among the current commercially available tests are ExoDx and Liquid CDx. The test ExoDxTM Prostate (IntelliScore) detects RNA from three genes (ERG, PCA3, and SPDEF) that have been linked to the development and progression of prostate cancer. The RNA is encapsulated in lipid membrane-coated structures called exosomes that are excreted by cancer cells into urine. FoundationOne Liquid CDx is an FDA-approved next generation sequencing-based in vitro diagnostic device that targets 324 genes utilizing circulating cell-free DNA (cfDNA) isolated from plasma derived from the anti-coagulated peripheral whole blood of cancer patients [80]. The latter test includes HPCa related genes. The detection of a mutation and, hence, of a positivity to the somatic test in the genes related to HPCa, must be confirmed in the germline. In this way, identifying the mutation in the HPCa susceptibility gene, the test allows an early diagnosis of HPCa.

## 7. Genetic Counselling, Guidelines for Genetic Test and Surveillance

Early onset of aggressive PCa combined with family history of PCa or other heritable cancers are strong predictors for a hereditary component arguing that those patients are candidates to undergo genetic testing.

To date, guidelines give no consistent recommendations about which patients, and at what stage of the disease, should undergo genetic testing [6]. In Table 1 we report a summary of the current guidelines on HPCa.

Men with highly aggressive PCa typically receive treatment whereas active surveillance is recommended for patients with low grade PCa and with non-metastatic PCa; therefore, they are unlikely to be harmed by their cancer during a period of observation. The much-debated topic concerns the characteristics that make a patient eligible for surveillance; indeed, different centers have proposed different criteria to guide clinicians in determining the eligibility of a patient for active surveillance. Commonly used criteria include low-grade and low-volume tumor on biopsy and low prostatic specific antigen (PSA) level [81]. 

PSA is a serine protease produced by both malignant and normal cells [82]. However, in healthy individuals, it is secreted into glandular ducts in concentrations million times higher than in plasma whereas, in PCa affected patients, a damage occurring in the secretory pathways leads to PSA leaking into the extracellular space [82]. Consequently, increased PSA level in serum can be observed, thus representing a sign of both inflammatory and neoplastic processes in the prostate [82]. A more recently used method to measure PSA is prostate health Index (PHI), which combines PSA, free PSA and p2PSA4. To date, compared to traditional PSA methods, the accuracy of PHI value still remains to be defined [82].

In Western countries, the widely accepted threshold value of PSA is 4.0 ng/mL. For value > 4.0 ng/mL, it is recommended to perform a biopsy to verify or exclude the presence of PCa. However, this cut-off value is not uniform across the ethnicities [82].

It is well known that the PSA test is widely used for PCa screening; however, it can also provide false positive data causing unnecessary prostate biopsies and inaccurate diagnosis of low-risk PCa. Indeed, careful studies support that a PSA screening every two years reduces the death rate for PCa of about 80% compared with annual screening while decreasing both the number of tests and the chance of a false-positive test by 50% and overdiagnosis by 30%. However, the various organizations declare in their guidelines that the ideal screening interval, is unknown. Indeed, The American Cancer Society recommends screening every two years screening for men aged ≥50 years and with a PSA level of <2.5 ng/mL and testing every year for PSA level ≥2.5 ng/mL. The AUA recommends biennial testing for men aged 55–69 years. The NCCN recommends 2- to 4-year intervals for men aged 45–74 years with PSA <1 ng/mL and 1–2 years for men with PSA ≥1 ng/mL. Recently, the US Preventive Services Task Force (USPSTF) suggests more observational studies are needed to evaluate the harms and the benefits of the different screening intervals [85].

The Gleason Score is a grade that the pathologist assigns after prostate biopsy or during trans-urethral resection for prostatic hyperplasia. It describes the aggressiveness of the cancer and directs the urologist to a definitive treatment or active surveillance of the patient [86].

Men with a personal history of Gleason ≥7 PCa with a family history of a *BRCA1/2* mutation, or one close relative with ovarian or breast cancer at age <50 years, or two relatives with breast, pancreas, or Gleason ≥ 7 PCa at any age, should undergo genetic counseling [6].Currently, the IMPACT multicenter observational study is evaluating the utility of performing these screenings, it being understood that men with age 40–69 are offered annual PSA and the threshold for prostatic biopsy is PSA > 3 ng/mL [30].

**Figure 3:**
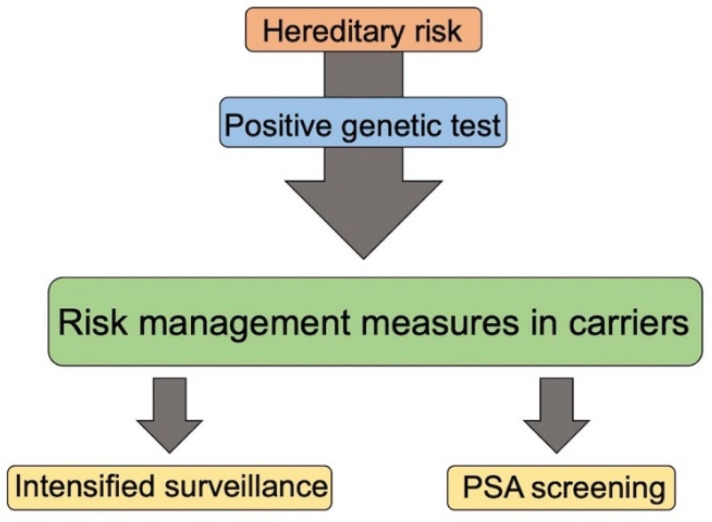
Management of unaffected family members with positive genetic test.

Regarding the surveillance to be carried out on unaffected men with mutations in DDR or MMR genes belonging to HBOC and LS families, there are no currently comprehensive guidelines regarding HPCa prevention. In a multicenter study concerning early diagnosis for men carrying *BRCA2* mutations, systematic PSA screening was recommended (Figure 3) [87].

The IMPACT study evaluated PSA screening in men with a known genetic PCa predisposition due to *BRCA1/2* mutations and found that a value of PSA >3 ng/mL is more strongly predictive in *BRCA* carriers than noncarriers [16,88].

Other authors reported that BRCA mutations and MMR deficiency and HOXB13 are associated with a higher Gleason score [67,76,89]. However, their clinical application, including their utility in screening programs, is as yet undefined and, in any case, none have been found sensitive and specific enough to replace the PSA measure [30].

## 8. Liquid Biopsy

The frequency of alterations appears to differ among genes involved in the DNA-repair pathways at different stages of the PCa. The evaluation of the results of NGS sequencing performed on tumor cells does not always give accurate results, since the evolutionary history of the sub-clonal lineages of tumor cells present in the sample may not provide those alterations that were formed later and, hence, may not offer a sufficient vision of current disease biology. Specific strategies have now evolved to overcome the issues of inter- and intra-tumoral heterogeneity and of the chronological changes occurring during the disease progression. Among them, liquid biopsy allows the evaluation of circulating cell-free DNA from blood samples [15]. 

Although tissue biopsy is the “gold standard” for diagnosing cancer, the removal of tumor tissue by surgery or, in some cases, through needle biopsy, is an invasive process. In addition, sometimes the difficulty of reaching the tumor can limit the ability of sampling. Moreover, several studies have indicated that the genetic information contained in the circulating tumor cells may provide important information on the tumor, such as the probability of response or relapse after treatment or on the response to a specific therapy. Thus, liquid biopsy may represent a promising and non-invasive examination to be performed [90,91].

Liquid biopsy involves the analysis of tumor material from readily accessible body fluid samples. The main utilized approaches include the analyses of circulating tumor DNA (ctDNA), RNA, including micro RNAs, proteins, and mitochondrial DNA, as well as circulating tumor cells, or extracellular vesicles. The analysis of ctDNA was evaluated in the definition for profiling tumor genomics in patients with metastatic prostate cancer. Particularly, patients with metastatic carcinoma displayed high ctDNA values, while the values were very low in patients with localized carcinoma [92]. Therefore, this analysis could make a positive contribution to the early diagnosis of HPCa, which is a more aggressive disease. Liquid biopsies have also emerged as an attractive strategy to study the PCa molecular setting in a slightly invasive manner. Although it has been widely used in the study of PCa, this strategy still needs to be implemented before being adopted in the routine clinical use [90,91]. In this context, a suitable and comprehensive NGS panel with HPCa associated genes for mutational analysis is still lacking and should be established.

To date, there are still several issues in the interpretation of tumor-profiling results. To this purpose, the American College of Medical Genetics has promulgated guidelines to aid laboratories in best reporting of findings in hereditary cancer genes. It is recommended that in the presence of an alteration in a hereditary cancer gene, the patient should be referred to a cancer genetics program for further evaluation [15].

## 9. Conclusions

HPCa remains an important clinical entity, with a spectrum of epidemiologic and genetic risk factors. Advances in NGS sequencing will allow new discoveries of PCa genetic predisposition. A more accurate knowledge of the mechanisms of HPCa predisposition could be brought by individualized PCa screening and treatment. As for the main cancer predisposition syndromes, including Lynch and HBOC, commercial NGS panels contain a large number of cancer susceptibility genes to detect mutations in patients with inherited cancer predisposing syndromes [46,93]. This useful approach has the advantage of being cost effective and to have a relatively feasible handling of raw data through validated bioinformatics pipelines; moreover, the detection of VUS, which are difficult to interpret in the clinical management, is quite limited [46,93]. Noteworthy, targeted sequencing of 94 cancer genes has also been recently used in probands with early onset/familial prostate cancer and has allowed successful identification of novel putative PCa predisposing germline mutations [94]. However, by multigene panels genetic testing, between 70–92% of patients (depending on the cancer syndrome), still remain mutation-negative or undiagnosed [93,95]. Thus, the use of Whole Exome Sequencing (WES) and Whole Genome Sequencing (WGS) strategies will be the preferred method in the near future when decreased costs and improved pipeline analyses will also make these strategies more suitable in the clinical setting. In this context, NGS methods will be useful even in searching for novel common variants conferring small to modest effect sizes by GWAS in patients with PCa predisposition [96]. Furthermore, approaches like RNA sequencing may allow the identification of genetic causes that are not recognizable by genomic DNA screening [93].

The liquid biopsy approach could allow not only an early diagnosis but also an analysis of the genetic tumor characteristics that are already present and that are relevant to providing the best therapy. For instance, molecular testing is able to identify patients who could benefit from PARPi treatment or platinum chemotherapy and to determine the cancer risk in family members. Current ongoing clinical trials may provide new indications on combinations of PARPi and immune checkpoint inhibitors with alterations in MMR and HR genes. To date, guidelines give no uniformed recommendations on which patients should undergo genetic testing and, at the same time, on the tests to be performed. Therefore, a clear policy regarding genetic testing could point to a more accurate active surveillance as a management strategy for patients with low-risk PCa.

## Figures and Tables

**Table 1:** Summary of the current guidelines on hereditary prostate cancer.

Johns Hopkins group (1998) [83]	One of the following criteria must be included for the consideration of familial prostate cancer (PCa): 1. Three or more first-degree relatives with PCa; 2. Three successive generations of PCa; 3. Two relatives with PCa diagnosed at age ≤55 years
American College of Medical Genetics (2015) [84]	Genetic testing should be considered if one of the following criteria is met: 1. Three or more first-degree relatives with PCa; 2. Two or more first-degree relatives diagnosed with PCa at age ≤55 years; 3. Gleason grade > 7 PCa and a family history of ≥2 individuals with breast, ovarian, or pancreatic cancer
American Society of Urology (AUA 2017) European Association of Urology (EAU 2019) [6]	Recommend offering germline genetic testing for BRCA1, BRCA2, ATM, PALB2, and FANCA to all patients with high risk or metastatic disease regardless of family history. For those patients with a lower-risk disease, germline genetic testing should be considered when: 1. There is a strong family history (brother or father or multiple family members diagnosed with PCa <60 years), 2. Known germline abnormalities and/or more than one family member with breast, ovarian, or pancreatic cancer (suggestive of BRCA2 mutations), 3. More than one family member with Lynch syndrome (LS)
National Comprehensive Cancer Network (2018) [22]	Recommend genetic testing of BRCA in PCa patients with one of the conditions listed below: 1. A history of Gleason grade ≥7 PCa regardless of age and ≥1 close relative with breast cancer (age ≤ 50 years) and/or invasive ovarian cancer; 2. Patients with prostate cancer (Gleason ≥ 7) who have two relatives with breast, pancreatic, or PCa (Gleason ≥ 7) diagnosed at any age; 3. A personal history of metastatic PCa (radiographic evidence of or biopsy-proven disease)
National Comprehensive Cancer Network (updated version 2019) [6]	States that. if next generation sequencing (NGS) is used, the panel must include BRCA1, BRCA2, ATM, CHEK2, PALB2, MLH1, MSH2, MSH6, and PMS2

## Data Availability

Not applicable.
